# Increase in Adult *Clostridium difficile*–related Hospitalizations and Case-Fatality Rate, United States, 2000–2005

**DOI:** 10.3201/eid1406.071447

**Published:** 2008-06

**Authors:** Marya D. Zilberberg, Andrew F. Shorr, Marin H. Kollef

**Affiliations:** * University of Massachusetts, Amherst, Massachusetts, USA; † Evi*Med* Research Group, LLC, Goshen, Massachusetts, USA; ‡ Washington Hospital Center, Washington, DC, USA; § Washington University School of Medicine, St. Louis, Missouri, USA

**Keywords:** Clostridium difficile–associated disease, hospital, costs, population epidemiology, dispatch

## Abstract

Virulence of and deaths from *Clostridium difficile*–associated disease (CDAD) are on the rise in the United States. The incidence of adult CDAD hospitalizations doubled from 5.5 cases per 10,000 population in 2000 to 11.2 in 2005, and the CDAD-related age-adjusted case-fatality rate rose from 1.2% in 2000 to 2.2% in 2004.

*Clostridium difficile*–associated disease (CDAD) represents a considerable public health hazard. In the United States, it is responsible for more deaths than all other intestinal infections combined (*1*). Incidence, hospitalizations, and deaths related to CDAD have been on the rise (*1*–*3*). Emergence of hypervirulent strains and in vitro resistance to third-generation cephalosporins and fluoroquinolones have also been reported (*4*). In view of these phenomena, it is unclear whether the recently reported 35%-per-year increase in CDAD-related deaths represents a rise in case-fatality rate or reflects increasing incidence of hospitalizations with this disease (*1*). We hypothesized the latter to be at least partially the cause. Given that 80% of all CDAD-related deaths occur in acute-care hospitals (*1*), we conducted a population-based analysis of CDAD-related adult hospitalizations in the period 2000–2005.

## The Study

We identified CDAD-related hospitalizations for 2000–2005 from the National Inpatient Sample data (*5*), available on the Healthcare Costs and Utilization Project Net website, administered by the Agency for Healthcare Research and Quality (*6*). The National Inpatient Sample is a stratified 20% sample of US community hospitals, and the data are weighted to provide national estimates (*5*). CDAD was identified by the presence of the International Classification of Diseases, 9th revision, Clinical Modification (ICD-9-CM), diagnosis code 8.45 (intestinal infection with *Clostridium difficile*), and the numbers of discharges per year were age stratified. We obtained censal and intercensal data (numerical and demographic characteristics of the US population from 2000 through 2005) from the US Census Bureau (*7*). On the basis of these data, we calculated age-specific hospitalization incidence rates and fitted linear models, using the least-squares method, to describe this age-specific growth. Finally, using the population-based CDAD mortality numbers in the report by Redelings et al. (*1*), we computed case-fatality rates for hospitalized CDAD patients for the 5-year period from 2000 through 2004.

The number of adults discharged from US hospitals with a CDAD diagnosis rose by nearly 160,000, from 134,361 in 2000 to 291,303 in 2005 (Table) (*6*). This 117% rise in CDAD discharges over a 5-year period equates to an ≈23% average crude growth annually. As a benchmark, we examined the changes in overall hospital discharges and discharges with CDAD as the principal diagnosis over the same period. We found the overall hospitalizations rose ≈1.3% annually (from 36,417,565 in 2000 to 39,163,834 in 2005). Although the absolute change in volume of cases for which CDAD was the principal diagnosis mirrored those in all CDAD admissions, the relative contribution of CDAD primary diagnosis to all CDAD cases remained relatively stable over time at ≈25%.

**Table:** Absolute numbers of adult hospitalizations with *Clostridium difficile,* by age group, United States, 2000–2005

Hospitalizations	2000	2001	2002	2003	2004	2005
18–44 y	14,738	15,001	18,747	19,393	22,168	25,662
45–64 y	28,280	29,527	39,421	43,290	50,898	61,757
65–84 y	69,018	74,010	98,148	105,404	122,875	147,675
>85 y	22,325	25,194	31,899	35,363	43,341	56,209
**All adult**	**134,361**	**143,732**	**188,215**	**203,450**	**239,282**	**291,303**

The numbers of adult hospital patients discharged with a CDAD diagnosis from 2000 through 2005 by age group are presented in the Table (*6*). The Figure illustrates the age-specific growth in CDAD incidence for the same period. The rate of increase in the incidence of CDAD was steepest in the >85 age group, with the slope for the linear trend 11.3 (95% confidence interval [CI] 7.6–14.9, p = 0.001), and ranged from 0.2 (95% CI 0.1–0.3, p<0.001) among the 18–44 age group to 4.8 (95% CI 3.2–6.0, p<0.001) among the 65–84 age group; the overall CDAD hospitalization incidence rose from 6.4 cases per 10,000 in 2000 to 13.1 cases per 10,000 in 2005. When published population-based CDAD mortality estimates were applied to the annual CDAD hospitalization volumes, the crude case-fatality rate rose from 1.2% in 2000 to 2.3% in 2004; age-adjusting the 2004 estimate resulted in a similar case-fatality rate of 2.2% (*1*).

**Figure:**
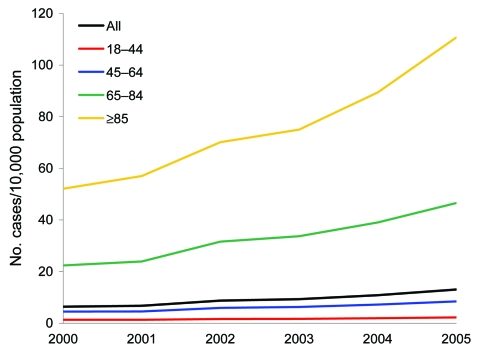
Changes in the age-specific *Clostridium difficile*–associated disease incidence rate per 10,000 population in the United States, 2000–2005.

## Conclusions

In our analysis we detected a 23% annual increase in CDAD hospitalizations in the 6-year period from 2000 through 2005. Moreover, the absolute number of CDAD hospitalizations more than doubled in all age groups except the youngest, for whom they increased by 74.1% over the study period. Additionally, we estimated that the age-adjusted case-fatality rate for CDAD hospitalizations nearly doubled from 1.2% in 2000 to 2.2% in 2004.

Our numbers help put in perspective the observed increasing mortality rates related to CDAD in the United States. The recent report by Redelings et al. noted an increase from 5.7 to 23.7 deaths with CDAD per million population from 1999 through 2004 in the United States, representing a 35% adjusted per annum increase (*1*). By observing a 23% per annum increase in the volume of hospitalizations with CDAD in the period 2000–2005, we demonstrate that at least half of the reported mortality increase with CDAD is due to an increase in the incidence of hospitalizations with this severe infection. Increased hospitalization may in turn be related to a simple increase in the overall volume of CDAD or reflect the increased virulence of the organism, leading to more cases of severe disease requiring hospitalization. We have also estimated that the unadjusted case-fatality rate did indeed increase from 1.2% in 2000 to 2.3% in 2004. While this doubling of deaths with CDAD is mirrored almost perfectly by the more-than doubling of CDAD admissions among all but the youngest age groups, who cumulatively represent 90% of all CDAD hospitalizations, age-adjusting the 2004 case-fatality estimate did not change it substantially. This finding indirectly confirms that the reported increase in CDAD deaths likely represents the effects of increased virulence of the organism (*1*,*4*).

Our analysis relied on ICD-9-CM coding to identify CDAD-related hospitalizations. Studies correlating the presence of the diagnostic code for CDAD to the presence of a laboratory confirmation of the disease have not suggested a clear over- or underdiagnosis trend in the administrative coding (*2*). However, the administrative nature of the data may have predisposed our case ascertainment to misclassification. Giving credence to our numbers, however, is the report by McDonald et al., who noted near-doubling of CDAD US hospital discharges, from 98,000 in 1996 to 178,000 in 2003 (*2*). Additionally, while exhibiting a similar absolute rise, CDAD primary diagnosis admissions as a fraction of all CDAD hospitalizations remained constant. Although it is possible that the observed rise in CDAD hospitalizations is due to changes in coding practices, evidence of an increase in microbiologic detection of this pathogen argues against this explanation for our observations (*8*).

The incidence in adult CDAD-related hospitalizations increased substantially in the period 2000–2005. In view of the aging US population, this rapid pace of growth is alarming. If this rate of rise, along with the increase in virulence and diminished susceptibility to antimicrobial drug treatments, persists, CDAD will result not only in a considerable strain on the US healthcare system (*9*,*10*) but also in rising numbers of deaths related to this disease (*1*). Allocation of public health resources aimed at prevention of CDAD is necessary to mitigate this growing epidemic. Research into the best preventive strategies, such as limiting the use of antimicrobial agents in both human disease and the food supply (*11*), is a public health imperative.
